# Evaluation of the Ecochemical Status of the Danube in Serbia in Terms of Water Quality Parameters

**DOI:** 10.1100/2012/930737

**Published:** 2012-05-03

**Authors:** Ljiljana Takić, Ivana Mladenović-Ranisavljević, Milovan Vuković, Ilija Mladenović

**Affiliations:** ^1^Faculty of Technology Leskovac, University of Niš, Bulevar Oslobođenja 124, 16000 Leskovac, Serbia; ^2^Tehnical Faculty in Bor, University of Belgrade, Vojske Jugoslavije 12, 19210 Bor, Serbia

## Abstract

The Danube is an international river passing partly through Serbia. The protection of the environment and sustainable use of water resources is a primary task that implies constant monitoring of the quality status and evaluation of ecochemical status of the water in the Danube basin. The investigation includes calculation of all-inclusive water quality by the Serbian water quality index (SWQI) method and an evaluation of eco-chemical status of the Danube water in terms of water quality parameters from the entry to the exit point along its course through Serbia in the year of 2009. The results show that the overall quality of the Danube water on the territory of Serbia corresponds to the descriptive indicator of “*very good*” water. According to the *Council Directive75/440/EEC*, the evaluation of the ecostatus, with slight deviation of individual parameters at Pančevo, corresponds to A1 category of the surface water quality intended for the abstraction of drinking water supplies in member states.

## 1. Introduction

The Danube River is 2857 km long and has a basin of 817000 km^2^. It connects the western and eastern Europe. The European blue highway runs through Serbia in a length of 588 km and has a catchment area of 102.350 km^2^. On its course through Serbia, the Danube extends from the three-border area of Serbia, Hungary, and Croatia to the mouth of Timok, where the borders of Serbia, Bulgaria, and Romania are, and then passes through vivid Vojvodina Plain and national parks of “Fruška Gora” and “*Đ*erdapska klisura.” In addition to natural wealth, there are numerous cultural and historical values that are dotted along its shores. The main tributaries of the Danube River in Serbia are the Tisa, the Sava, the Tamiš, the Morava, the Nera, and the Timok [1].

Having in mind that the Danube River is one of the most valuable natural water resources in Serbia, a special attention must be paid to its evaluation, pollution, and protection problems. In the structure of registered Danube polluters, industry, agriculture, settlements, and energy dominate. From the economic and population point of view, the most developed part of Serbia is located along the river banks, so the industry is considered to be the biggest polluter of the Danube [2]. Although Pančevo belongs to the wider industrial zone of Belgrade, it stands out as an ecological “black spot” in the Danube Region because it represents a great hazard for the environment. Chemical industry complexes—refinery, petrochemical industry, and fertilizer plant in Pančevo—are located in the southern zone of the town, almost at the very bank of the Danube and represent factories with great impact on the environment [3]. The eco-chemical status of the Danube is a subject of great interest both locally—in every country the Danube passes through—and internationally. The International Commission for the Protection of the Danube River (ICPDR), established in 1998 in Vienna, has a great importance and takes part in the implementation of the Danube River Protection Convention [4]. ICPDR has a special role in forming an accident emergency warning system, a transnational monitoring network for the Danube water monitoring, and an information system on the Danube. Serbia has taken an active part in current international projects in the field of environmental protection in the Danube Region [5]. Mihić et al. [6] promote the importance of environmental protection in the Danube River Basin suggesting that, in order to manage the sustainable development strategy, it is necessary to conduct an all-inclusive analysis on the current state. The protection and improvement of environment are supported by the monitoring data on surface water quality status along with the changes of eco-chemical status [7]. Long-term changes of parameters, indicators of the Danube water quality, differ in time and space. Živadinović et al. [8] point out the difference in the water quality at the entry (Bezdan) and exit (Radujevac) profile for the period from 1992 to 2006 as well as constant improvement and the satisfactory trend of eco-chemical status of the Danube River on the territory of Serbia.

The results of this investigation are important from the aspect of environmental protection and as a basis for further monitoring of changes in this aquatic system together with development of the regional transboundary cooperation through transnational ecological network.

## 2. Methodology

The Republic Hydrometeorological Service of Serbia (RHSS) carries out a systematic monitoring of quantitative and qualitative characteristics of the surface and groundwater in order to ascertain, analyze, and monitor the water regime on the Serbian territory. The extent, type and frequency of the waterway quality control are stipulated by the Program for water quality testing according to the Water Act, and the Regulation on the systematic water quality testing passed by the Government of the Republic of Serbia. According to the adopted methodology, the basic physicochemical indicators are tested once a month. Additional physicochemical parameters are checked at least four times a year, while metals and other hazardous substances are controlled three to twelve times a year, depending on the economic importance of the given part of the waterway. Total radioactivity is measured two to seven times per year in more important profiles. The results of the implemented monitoring of the water quantity and quality are reflected in a significant number of information (date and time of sampling, water level, and flow rate at the sampling time, quality parameters, values) collected in the Data Fund of the RHSS [9].

The starting point for the monitoring of quality and eco-status of the River Danube water in Serbia is the Data Fund of the RHSS for the year 2009 and the selection of the quality parameters for the index method. The investigation on the state of Danube water quality includes sixteen hydrological measuring stations at distances given from the river mouth:

1. Bezdan (entry point)—1425.59 km
2. Apatin—1401 km
3. Bogojevo—1367.4 km
4. Bačka Palanka—1298.6 km
5. Novi Sad—1254.98 km
6. Slankamen—1215.5 km
7. Senta—1189 km
8. Zemun—1174 km
9. Pančevo—1154.6 km
10. Smederevo—1116.0 km
11. Banatska Palanka—1076.6 km
12. Veliko Gradište—1059.2 km
13. Dobra—1021 km
14. Tekija—956.2 km
15. Brza Palanka—883.8 km
16. Radujevac (exit point)—852 km.

In this evaluation three hydrological measuring stations were selected to represent the three main points of the Danube River on its course through Serbia—entry point (Bezdan), medium point (Pančevo), and exit point (Radujevac).

At the sampling point, the water temperature was measured and pH value determined according to SRPS H.Z1.111 method; biochemical oxygen consumption (BOD-5) was determined by EPA 360.2 method, suspended solids according to 13.060.30 SRPS H.Z1.160 method, phosphates according to standard analytical method APHAS AWWA WEF 4500-), total nitrogen oxides according to SRPS ISO 5664 method, while the estimated number of coliform bacteria (*E. coli*) per liter was determined 48 hours after incubation at 37°C [9].

**Figure 1:**
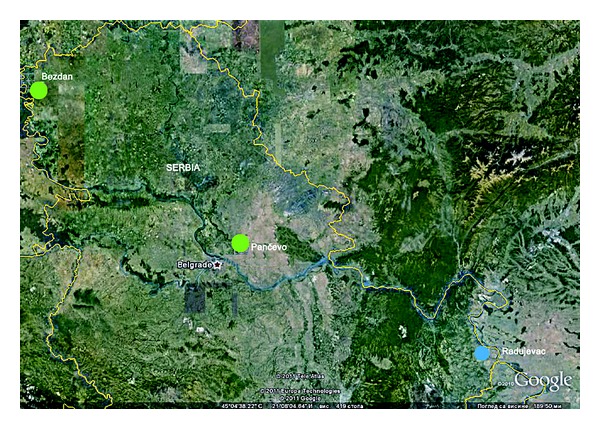
Map of the Danube water quality testing profiles.

Map of the Danube water quality testing profiles along with their quality indicators represented by colors is shown in Figure 1.

The study of the surface water as a complex multi-component system depends on the knowledge and application of facts, principles, and methods from chemistry, physics, geology, hydrology, meteorology, mathematics, and other sciences with the purpose of solving the essential ecological problems. The particularity and complexity of the surface water chemical composition and of quality indicators (representing the effects of various dissolved substances such as mineral and organic matter, gases, colloids, suspended particles, and microorganisms, present in water through natural or artificial processes) stress the importance of applying quality index methods for their assessment by identifying a common factor which encompasses quality as a whole.

The Serbian Environmental Protection Agency [10] has developed an indicator of the environment for the area of water intended for reporting to the public, experts, and decision makers (local government, state agencies). The indicator is based on the water quality index (WQI) method [11] according to which the ten selected parameters (oxygen saturation, *E. coli*, BOD-5, pH value, total nitrogen oxides, orthophosphates, suspended matter, ammonium, temperature, and conductivity) aggregate in the composite indicator of the surface water quality. These parameters have the quality (*q*_*i*_). The share of each of the ten selected parameters does not have the same relative significance in the overall water quality; therefore, each of them was allocated a specific weight (*w*_*i*_) according to the share in jeopardizing the quality. By summing their products (*q*_*i*_ × *w*_*i*_), index 100 is obtained as the ideal sum for all parameters. Depending on the number of points of the individual parameters, given water will be allocated a number of index points ranging from 0 to 100. In case of missing data for an individual parameter, the value of arithmetically determined WQI is corrected by multiplying the index value by 1/*x*, where *x* is the sum of arithmetically measured weights of available parameters [12].

The classification system of the surface water quality description according to the Serbian water quality index (SWQI) represents a method for the evaluation of quality using a group of selected parameters, and so by applying this method, an overall evaluation of the surface water quality status can be obtained. SWQI indicators of the surface water quality were determined in comparing the quality parameters of Serbian classification with those of the original WQI method. Determination of the water quality using a single index number is a simpler approach than comparing the measured individual indicators of the water quality parameters with the reference values [13]. The adopted classification criteria of the descriptive quality indicator and determination of the surface water class based on the calculated value of SWQI index number are given in Table 1 [10].

Quality indicators of the surface water are classified, in compatibility with the existing classification, according to their purpose and the purity level.

- Excellent—the water in its natural state that can be used to supply settlements with water, as well as in food industry (by using filtration and disinfection methods), while as a surface water it can be used for cultivation of precious fish species.
- Very good and good—the water in its natural state that can be used for swimming and recreation, water sports, for breeding of other fish species, or by using modern purification methods, for water supply systems, and in food industry.
- Poor—the water that can be used for irrigation or, after being treated with modern methods, for industries other than food industry.
- Very poor—the water that has an adverse effect on the environment and can only be used after special treatment methods (purification).

The SWQI indicators of the surface water quality are represented by colors on waterway maps indicating the corresponding control profiles as in Table 2 [10].

The European Union has defined its long-term policy in the water domain when the European Parliament and the EU Council adopted the Water Framework Directive EU/WFD–2000/60/EC on October 23, 2000. EU/WFD is the most important legal instrument in the water domain and a precondition for successful realization of the concept of integrated environment management. Specific conditions regarding the implementation of the adopted policy of the sustainable use and protection of water are formulated in the directive. The primary aim of the framework directive is bringing all natural waters into “good status,” that is, providing good hydrological, chemical, and ecological status of water bodies [14]. The catchment-based approach of the EU/WFD, coupled with the requirement to achieve “good status” for receiving waters and the requirement for stakeholder involvement, has introduced much needed flexibility into the process which is expected to lead to better and more cost-effective solutions to water quality problems [15].

The basic principles contained in the directive will be implemented not only in the EU member states but also in candidate countries. Obviously, this document will be the basis for the realization of defined plans for water resources management within the international river basins in Europe. Assessment of compliance in 2015 is likewise a challenging task. It can be carried out at a range of levels from simple expert judgment, using a few key parameters combined with empiric experience, to complicated modeling involving numerous parameters [16]. That means Serbia must initiate the harmonization of its national legislation, regulations, standards, and institutions in the water and environment domain with those of the European Union [17].

As regards the determination of the surface waters quality from the aspect of its suitability for water supply, SWQI method is comparable to the *Council Directive 75/440/EEC*, which refers to the required quality of surface water intended for human consumption in member states [18].

According to this directive, Annex I, with respect to the limit values of the quality parameters, the surface waters are categorized into three categories with suggested standard treatment methods for transforming surface water into drinking water.

(*A*1) Simple physical treatment and disinfection, for example, rapid filtration and disinfection.
(*A*2) Normal physical treatment, chemical treatment, and disinfection, for example, prechlorination, coagulation, flocculation, decantation, filtration, disinfection (final chlorination).
(*A*3) Intensive physical and chemical treatment, extended treatment and disinfection, for example, chlorination to break point, coagulation, flocculation, decantation, filtration, adsorption (activated carbon), disinfection (ozone, final chlorination).

These groups correspond to three different surface water qualities according to their physical, chemical, and microbiological characteristics set out in the table given in Annex II. Surface water having physical, chemical, and microbiological characteristics falling short of the mandatory limiting values corresponding to treatment type A3 may not be used for the abstraction of drinking water. However, such lower-quality water may, in exceptional circumstances, be used if adequate procedures (including blending) are applied in order to bring the quality characteristics of the water up to the level of the quality standards for drinking water (European Council Drinking Water Abstraction Directive).

## 3. Results and Discussion 

The selected parameters show physical, chemical, and microbiological characteristics of the water, and they give a summary of the River Danube water quality by calculated value of the SWQI index number. Based on the results of analyses of monthly samplings, the average value was calculated for the corresponding SWQI parameters of water quality for every measuring station annually, and from the median of SWQI indices array of the selected measuring stations the overall SWQI water quality index for the River Danube was calculated for the year 2009.  Table 3 shows the water quality at the selected measuring stations along the River Danube, expressed in SWQI values.

The calculated values of SWQI for the selected measuring stations in 2009 show that the River Danube quality was in range from 81 to 88 corresponding to descriptive indicators of “*good*” and “*very good*” water. At the entry profile Bezdan the SWQI index value was 82, and at the exit profile Radujevac 88, while the lowest index value was 81 at the measuring station in Pančevo downstream of Belgrade. The overall quality of the River Danube water on Serbian territory, determined by the median of the array of mean index values, amounted to SWQI = 83.6 for the one-year period, which corresponds to the descriptive indicator of “*very good*” water.

The evaluation of the eco-chemical status is based on a comparative analysis of the results, that is, the minimum, maximum, and median value of the array of the selected parameter indices for water quality, with standard parameter values defined by the *Council Directive 75/440/EEC* as the required quality for the surface water intended for abstraction of drinking water in member states (Table 4).

The River Danube water temperature in winter-summer period ranges from 3.2°C to 25.3°C, which does not represent extreme changes of this parameter as a condition of maintaining water life, so that the average value is lower than prescribed for A1 category of river water as given by the *Council Directive 75/440/EEC*. The Danube has pH value of the water from weak alkali (7.1 min Radujevac) to weak acid (8.7 max Bezdan), which is common for river waters. The pH value defines solubility and biological availability of chemical compounds of nitrogen, phosphorus, carbon, and heavy metals. Waters that are very acid in character have an adverse effect on organisms in the aquatic ecosystem. The average pH value determines the measure of water acidity, indicating a certain level of organic pollution of the water, but still within the limits of A1 category. The values for conductivity show a low concentration of ions in the water, ranging from 248 *μ*S/cm (min Radujevac) to 518 *μ*S/cm (max Bezdan), and they are less than allowed for the river water of A1 category. The low level of oxygen saturation is a sign of possible pollution, which is not the case of the Danube because the values at all measuring stations are higher than 70%, which is the limit value for A1 category and the life sustainability of the water. The results of BOD-5 determination at the measuring stations Bezdan and Pančevo indicate a presence of biodegradable organic matter and classify the water into the A2 category, while the value of 1.9 mg/L at Radujevac indicates a decreasing trend of biological consumption of oxygen and an improvement of water quality. Suspended matters refer to the content of organic and inorganic pollutants in the water, and they are plentiful at Pančevo (78 mg/L) with obvious tendency to decrease by the Danube exit profile (30 mg/L), but still remaining higher than A1 category. The total nitrogen oxides and orthophosphates as an indicator of pollution by chemical industry are correlated showing a negative trend towards the exit profile of the river and a negligible deviation from A1 category. Phosphorus is considered to be the most critical growth factor in the water bodies because the dissolved phosphate is absorbed by the plants and passed on to animals in the food chain. The increase of the phosphates concentration above the natural level brings about eutrophication, which destroys the structure of the natural aquatic ecosystem, and loss of biodiversity. Ammonium ion strongly deviated from the limiting values for concentration for A1 category, but due to mildly acid character of the River Danube, water it remains within the A2 category. The presence of coliform bacteria (*E. coli*) is an indicator of the highest sanitary contamination of water at Pančevo, ranking it into A3 category, but the values are significantly lower toward the exit flow of the Danube from Serbia. The analysis of the results shows noticeably better quality at the exit profile as opposed to the entry profile of the Danube into Serbia.

## 4. Conclusions 

The European Union gives great significance to the protection and preservation of water resources and environment, treating them as the base of sustainable development in the 21st century. Therefore, the EU has decided not to leave such an important issue to autonomous decisions of individual countries within the union but to make a unique, coherent strategy for the environment protection and water management. The strategy includes integrated treatment of water problems, on one side, and environmental protection, on the other side, so that the legislative, technical, and economic approach in different countries must be harmonized. Water resources are considered to be the most important segment of the environment, and protection of natural environment is unthinkable without adequate protection of water which includes water monitoring, water classification, and regulation of water quality standards. This investigation shows that by use of SWQI method and a comparison with the standard values defined, by the *Council Directive 75/440/EEC* as the required surface water quality intended for abstraction for drinking water distribution in member states, a comprehensive evaluation of the water quality and eco-chemical status of the Danube can be obtained. The overall water quality of the Danube in Serbia corresponds to the descriptive indicator of *“very good”* water and meets the requirements of A1 category of the Council Directive 75/440/EEC for the observed one-year period. The applied SWQI method and the evaluation results should be regarded as a contribution of the Serbian Republic to European integrations in the field of environmental protection by implementation of WFD at the national level.

## Figures and Tables

**Table 1:** Classification of the surface water by the SWQI method.

	Serbian water quality index (SWQI)				
	Excellent	Very good	Good	Poor	Very poor
	100–90	89–84	83–72	71–39	38–0
WQI	85–84		74–69	56–44	51–35

**Table 2:** The SWQI surface water quality indicators.

Serbian water quality index		
Index (range)	Descriptive indicator	Color
100–90	Excellent	Dark blue
84–89	Very good	Light blue
72–83	Good	Green
39–71	Poor	Yellow
0–38	Very poor	Red
No data available		White

**Table 3:** SWQI water quality along the River Danube for the year 2009.

Parameters (unit)	(1) Bezdan	(2) Pančevo	(3) Radujevac
Temperature (°C)	13.2	14.6	15.5
pH value	8.3	8.2	7.7
Conductivity (*μ*S/cm)	411.8	398.3	372.6
Oxygen saturation (%)	97.7	95.7	93.2
BOD-5 (mg/L)	2.2	2.3	1.9
Suspended matter (mg/L)	32.4	31.6	9.8
Total nitrogen oxides (mg/L)	1.891	1.332	0.983
Orthophosphate (mg/L)	0.044	0.047	0.200
Ammonium (mg/L)	0.08	0.09	0.12
*E. coli* (u 100 mL)	11498	18525	636

SWQI	82	81	88
SWQI medium		83.6	

**Table 4:** Parameters of the Danube River at selected measuring stations along the course through Serbia.

	Temperature (°C)	pH	Conductivity (*μ*S/cm)	Oxygen saturation (%)	BOD-5 (mg/L)	Suspended matter (mg/L)	Total nitrogen oxides (mg/L)	Orthophosphate (mg/L)	Ammonium (mg/L)	*E. coli* (n/1l)
The Danube River, the entry profile—measuring station Bezdan										

Min.	3.2	8.0	334	79	1.0	6	1.091	<0.010	0.03	500
Max.	22.4	8.7	518	130	4.5	54	3.433	0.067	0.17	24000
Medium	13.2	8.3	411.8	97.7	2.2	32.4	1.891	0.044	0.08	11 498

The Danube River—measuring station Pančevo										

Min.	3.3	7.9	340	82	1.1	8	0.721	0.013	0.02	2100
Max.	24.2	8.6	465	128	4.2	78	1.922	0.070	0.16	24000
Medium	14.6	8.2	398.3	95.7	2.3	31.6	1.332	0.047	0.09	18 525

The Danube River, the exit profile—measuring station Radujevac										

min	3.9	7.1	248	74	1.5	2	0.228	0.053	0.02	<200
max	25.3	8.1	428	132	3.2	30	1.618	0.550	0.57	<2000
medium	15.5	7.7	372.6	93.2	1.9	9.8	0.983	0.200	0.12	636

Council Directive 75/440/EEC concerning the quality required of surface water										

A1	22	6.5–8.5	1000	>70	<3	25	1	0.4	0.05	20
A2	22	5.5–9	1000	>50	<5	—	2	0.7	1	2 000
A3	22	5.5–9	1000	>30	<7	—	3	0.7	2	20 000
